# Cesarean Section Rates and Mobile Health’s Role in Equitable Access to Prenatal Care

**DOI:** 10.3390/ijerph23030288

**Published:** 2026-02-26

**Authors:** Nicole Person-Rennell, Patrick Rivers, James Hollister, Alicia Dinsmore, Nicole Bratsch, Judith Ortiz, Kristen Rundell, Karen Lutrick

**Affiliations:** 1 Department of Family and Community Medicine, University of Arizona, Tucson, AZ 85711, USA; npersonrennell1@arizona.edu (N.P.-R.); aliciadinsmore@arizona.edu (A.D.); nicolebratsch@arizona.edu (N.B.); judyortiz@arizona.edu (J.O.); kristenrundell@arizona.edu (K.R.); klutrick@arizona.edu (K.L.); 2 Department of Community, Environment, and Policy, University of Arizona, Tucson, AZ 85724, USA; jameshollister@arizona.edu

**Keywords:** mobile clinics, mobile healthcare, prenatal care, cesarian sections, obstetric health indicators, NTSV, free and charitable clinics

## Abstract

**Highlights:**

**Public health relevance—How does this work relate to a public health issue?**

- Cesarean section (CS) rates have risen globally, and while an often lifesaving and necessary intervention, CS deliveries increase future maternal/neonatal risks and are costly to both patients and healthcare systems.
- This work examines CS rates in the setting of a free mobile health clinic for uninsured patients.

**Public health significance—Why is this work of significance to public health?**

- The provision of maternity care in a mobile healthcare setting has not been significantly evaluated in the literature, and this work would add data regarding the quality of maternity care provision using standardized outcomes in the setting of maternity care, specific to mobile healthcare.
- This work examines CS rates in a free mobile health clinic for uninsured patients to examine quality obstetric metrics and evaluate if this clinical work is consistent with low/at goal CS rates.

**Public health implications—What are the key implications or messages for practitioners, policy makers and/or researchers in public health?**

- These findings suggest that access to free prenatal care through a mobile health delivery model may contribute to favorable obstetric outcomes among uninsured individuals and have implications for addressing maternal and neonatal health inequities among those who face multiple barriers to receiving adequate prenatal care.
- Supporting mobile clinics targeting uninsured or other vulnerable groups may provide a method of meeting national CS targets and average rates in a higher-risk population.

**Abstract:**

Cesarean section (CS) rates have risen globally, and while an often lifesaving and necessary intervention, CS deliveries increase future maternal/neonatal risks and are costly to both patients and healthcare systems. The U.S. Department of Health and Human Services has set a national low-risk pregnancy CS (NTSV) target of 23.9% under the Healthy People 2030 initiative. This analysis compares NTSV rates of uninsured patients receiving prenatal care from a mobile clinic to the national target and also compares overall mobile health CS rates with national and state CS rates. Through reviewing 5 years of electronic medical records, we calculated an NTSV CS rate of 25.0% among our University of Arizona Mobile Health Program prenatal patients, an uninsured and medically vulnerable patient group. This rate is similar to both the most recent Arizona state average of 23.4% and the national target of 23.9%. The MHP total CS rate is 26% over our study period, which is less than the most recent National and Arizona rates of 32.3% and 29.0%. These findings suggest that access to free prenatal care through a mobile health delivery model may contribute to favorable obstetric outcomes among uninsured individuals and have implications for addressing maternal and neonatal health inequities among those who face multiple barriers to receiving adequate prenatal care.

## 1. Introduction

Rates of Cesarean section (CS) have significantly increased around the world in recent decades, rising approximately 19% from 1990 to 2018 [1]. The most recent data indicate that the global rate now averages approximately 21% and is predicted to continue rising [1]. This trend is concerning when considering the World Health Organization (WHO) recommends that population-based CS rates should not exceed 10–15% [2]. In a more local context, CS rates in 2021 were at 32.1% in the U.S overall, and there are significant racial/ethnic disparities with national rates for black pregnant people at 36% [3,4].

Notably, disparities have also been recorded between Hispanic vs. non-Hispanic patients living on the US-Mexico border. A study of birth certificates from 2015 found that low-risk, nulliparous Hispanic women living along the US-Mexico border had higher CS delivery rates (21.1%) when compared to non-Hispanic white women (16.5%) living in the same area [5]. These disparate rates between Hispanic and non-Hispanic white women were also found in California and the US overall in the years between that study and the beginning of the pandemic [6,7,8], as well as during the COVID-19 pandemic [9].

One of the issues of most concern about the increasing CS rate is that many CSs are performed without clear maternal indications [10]. CS procedures, although lifesaving in many cases, are associated with a higher healthcare cost burden and more short- and long-term health risks for both mother and baby when performed unnecessarily [11]. These include increased risk in future pregnancies of uterine rupture, abnormal placentation, and preterm birth [12].

Though data specifically about uninsured prenatal patients in the U.S. is scarce, there are documented disparities between the percentage of privately and publicly insured versus uninsured patients who undergo a CS [13,14,15,16]. One study found that the odds of receiving a CS procedure in the U.S. are 0.7 and 0.92 times lower, respectively, when comparing uninsured mothers to privately insured and publicly insured mothers [13]. The authors suggest that the disparity may be attributable to financial incentives, given that the reimbursement for CS is typically stronger in populations with public or private insurance rather than uninsured groups. They postulate that providers may have an inherent but unacknowledged preference for performing CS in insured women. Additionally, they note lack of access to needed, medically indicated CS in uninsured women may decrease this rate.

Given this background, it is important to understand local rates of CS and other quality metrics, including pregnancy complications, as well as how and why these metrics vary between uninsured, publicly insured, and privately insured groups. Even though the WHO recommends CS rates between 10 and 15%, Arizona’s rates in the most recently available year (2023) were 28.70% [4], with differences between groups of patients that should be understood and addressed in order to ensure that adequate health care is being provided to all groups.

As a doctor’s office on wheels, our University of Arizona Mobile Health Program (MHP) addresses the gap in outpatient prenatal care for uninsured and underinsured patients in Southern Arizona. Staffed by family medicine clinicians and financially supported by a number of entities, including the University of Arizona, Banner University Medical Group, and grants from several funders, including local government agencies, the March of Dimes, and non-profit organizations, the MHP offers comprehensive prenatal care at no cost to patients. Patients are recruited via word of mouth and outreach by community partners where the unit parks in order to provide care. MHP patients experience structural barriers to care, including the ability to pay for services, immigration status, and limited English-speaking abilities. The MHP seeks to increase access to medical care for these patients through its free prenatal clinics and primary care clinics.

The MHP is one of a dozen mobile clinics in the United States providing such comprehensive prenatal care. While some evidence is available suggesting that mobile clinics have the potential to increase access to prenatal care [17,18], very limited data have been published on the effect of mobile prenatal care on delivery outcomes, with no consistency in outcomes measured or conclusions drawn [17,19]. Given the paucity of research on this topic and the uniqueness of this approach to prenatal care, this appears to be a meaningful gap in the literature. The study reported here is a retrospective analysis of MHP prenatal patients’ CS rates. Findings suggest that CS delivery rates for our MHP patients are below the overall state and national average CS rates. These findings support mobile prenatal care as an appropriate medical service model for addressing health inequities among uninsured patients who may otherwise not have access to care.

## 2. Materials and Methods

Data were extracted from a chart review of the MHP electronic medical record (EMR, OneTouch EMR Version 3.0, rev 11-28945, Dallas, TX, USA) into a prenatal database to create a study sample for each of the study years 2019 (start of EMR use in April 2019) through 2024. The sample was composed of patients who were served by the MHP and delivered at the program’s affiliated/partner hospital. Vaginal deliveries were provided by a practice group of family medicine physicians, half of whom also provided care in MHP, and surgical deliveries were provided by obstetrician colleagues in the same institution. Delivery records were accessed through a chart review of the affiliated hospital’s EMR. Patients were excluded if they (1) had only one appointment with the MHP before week 34 of pregnancy (n = 66), as this denoted a transfer of care, for example, in the setting of becoming insured or requiring Maternal–Fetal Medicine specialist care (e.g., for multi-fetal gestation); and (2) delivered at hospitals other than the partner hospital.

Data for included patients were summarized using descriptive statistics, including demographic characteristics of the patients. Race and ethnicity data is optionally reported by patients in the MHP, with many patients opting out of providing this information.

Two specific CS rates were calculated for each of the study years: (1) rates of nulliparous, term, singleton, vertex (NTSV) pregnancies delivered by CS [4] and (2) total CS rate, the percentage of all live births that were delivered by CS [4]. These two specific CS rates were then compared to CS delivery quality standards and rates at national and state levels.

On the national level, the CS rates of MHP patients were evaluated from two perspectives: (1) by comparing the MHP patients’ NTSV CS rates to national standards; and (2) by comparing the MHP Prenatal Care Program patients’ NTSV and total CS rates to state and national rates [4]. The most recently available data for state indicators were used for comparison for NTSV and total CS rates, and one-sided Poisson Exact Tests were used to calculate an upper 95% confidence bound for the total and annual CS and NTSV rates. There were no available data on overall CS rates at the individual local hospitals. We calculated 95% confidence intervals via the Poisson test function in R (R Core Team, Vienna, Austria, version 4.5.2) [20].

## 3. Results

The MHP total CS rate for 2019–2024 was 26.0% (95% upper confidence bound: 31.1%), ranging from a low of 21.4% in 2021 (95% upper confidence bound: 37.4%) to a high of 33.3% in 2020 (95% upper confidence bound: 50.0%). National total CS rates from 2019 to 2024 ranged from 31.7 to 32.3%, with the most recent total CS rate available in 2023 at 32.3%, higher when compared to the MHP total CS rate for 2023 at 29.2%. For Arizona, the most recent data publicly available shows the 2023 state-wide total CS rate is 29.0% compared to the 2023 MHP total CS rate of 29.2%. The range for Arizona for the study period with total CS rates was 27.5–29%.

Between 2019 and 2024, the University of Arizona MHP provided prenatal care for 331 patient deliveries, including 120 deliveries among NTSV patients. During this period, the overall mean NTSV CS rate among MHP patients was 25.0% (95% upper confidence bound: 33.9%), which is near the U.S. Department of Health and Human Services (DHHS) Healthy People 2030 target of 23.9%. This is below all national NTSV CS rates over the study period (range 25.6–26.6). This rate is similar to Arizona NTSV rates over the study period (range 21.9–23.4%).

Table 1 presents the demographic characteristics of the MHP patients included in this analysis. The majority of patients (61.3%) identified as Latinx, reflecting the demographics of the population served in Southern Arizona. Race and ethnicity data are underreported by MHP patients for a variety of reasons, including confusion over racial categories when ethnically Latinx, as well as fear of reporting due to immigration status. 46.8% and 2.4% of patients had a pre-existing medical condition or mental health condition, respectively. This includes 43.3% and 0.8% among NTSV patients. Patients averaged 8.6 visits with the MHP, with NTSV patients averaging 8.9 visits.

**Figure 1:**
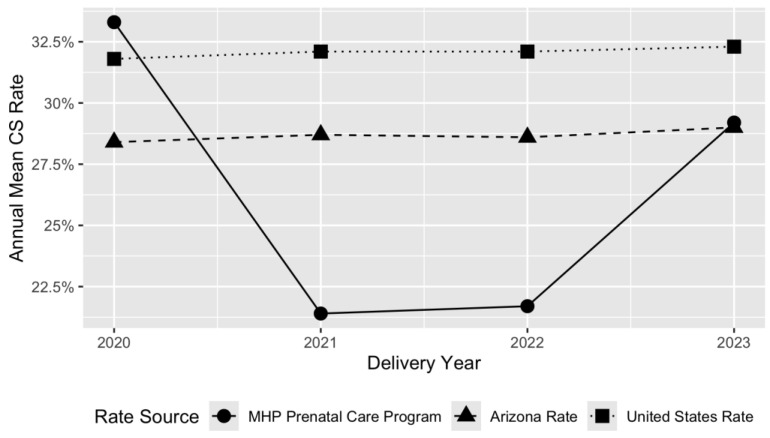
Comparison of average total CS rate between the MHP Prenatal Care Program, Arizona, and the United States, from 2020 to 2023.

**Figure 2:**
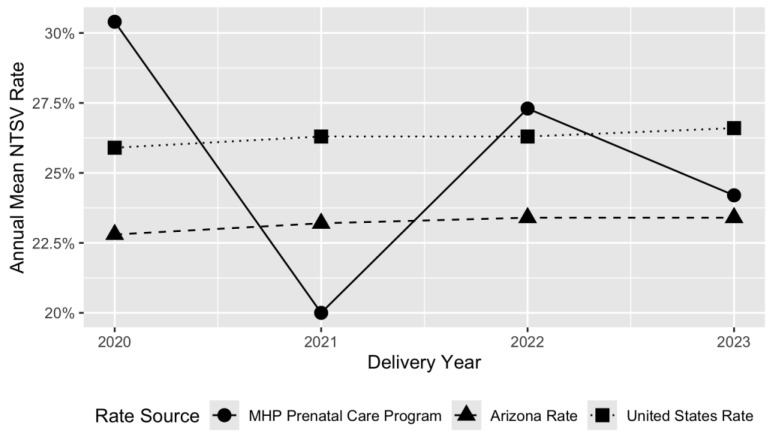
Comparison of average NSTV rate between the MHP Prenatal Care Program, Arizona, and the United States, from 2020 to 2023.

Figure 1 and Figure 2 illustrate MHP’s CS rates over time, comparing NTSV and total CS rates against Arizona state rates and national averages.

## 4. Discussion

The MHP provides a model of continuity of prenatal care for access to care for uninsured pregnant people in Southern Arizona, facing significant barriers. Our results indicate that CS rates for patients cared for in our MHP are similar to national quality targets and lower than the overall CS national and state averages. This suggests that free mobile prenatal services can improve maternal care for underserved patients who are otherwise unlikely to afford or access care without the MHP.

Different CS/obstetric delivery outcome quality metrics were compared in this study to ensure comparison of local data to nationally relevant metrics and reduce potential bias that would occur if only one standard were used. Evaluating differences between the NTSV rate and the total CS rate allows for evaluation of both a low-risk, important quality indicator in obstetric care and the total CS rate, which provides a widely available and comparable metric to national trends. NTSV rates are important to examine for removing the impact of previous uterine surgery (hysterotomy), which is not an exclusion criterion for the total CS rate and increases risk for surgical delivery. Finally, the CS rate used by the CDC is a frequently referenced, easily calculated rate that allows for comparison of data over recent years.

While not specifically studied, it is likely that components of MHP’s success in maintaining goal CS rates include providing linguistically and culturally concordant care, using community partnerships, and clinical care with a team of providers that works across different clinical settings to provide continuity of care. With contacts at over 80 non-profits, churches, and community organizations, the MHP relies on community partners as a critical link to connect uninsured pregnant individuals to the clinic and also with needed resources for those individuals. Continuity of care from prenatal care to delivery is possible because all of the prenatal providers at the MHP also deliver MHP patients at the local partner hospital. In the case of need for CS, the MHP family medicine doctors at the hospital facilitate the transfer of care to the obstetrician team.

Study limitations include a relatively small sample size and limited availability of current (2024–2025) national and state-level comparator rates, as they have not yet been released to the public, and as the clinic began using an electronic medical record only in mid-2019, the period for the current examination begins in 2020. This means that we are unable to compare outcome data to before the COVID-19 pandemic. While rates of all surgeries, particularly elective surgeries, were reduced early in the pandemic after the US Centers for Medicare and Medicaid Services moratorium in order to improve hospital capacity to care for COVID-19 patients [21], the overall rate of CS did not change as hospital COVID-19 burden increased throughout the pandemic [9]. This is likely due to labor not being a routine follow-up condition requiring hospital access. Despite our inability to compare CS and NSTV rates in our program to pre-pandemic levels, the tracking of data through 2024 allows for some understanding of how things changed as the pandemic progressed and after its end. An additional limitation to the current analysis is that hospital-level practices and individual provider decision-making can influence CS rates independent of prenatal care quality, and such factors are difficult to measure. As noted above, the lower rate of CS in our patients compared to national and state rates may be partly due to the lower financial incentives for performing CS in uninsured patients. However, our partner hospital is staffed by salaried physicians whose compensation is not dependent on billing rates. Finally, patients who delivered outside of the hospital system affiliated with the MHP are not included in this analysis due to a lack of access to other health systems’ EMRs and delivery outcomes.

Despite the apparent positive results of our study, the role that mobile healthcare plays in addressing inequities in obstetrical outcomes for both insured and uninsured patients requires further evaluation. Aggregation of patient outcomes across mobile clinics nationally would be beneficial.

## 5. Conclusions

Our findings suggest that free, mobile prenatal care provided to uninsured, medically vulnerable patients achieves CS rates comparable to state and national rates and national targets. These CS outcomes are significant given that our MHP prenatal clinic is providing care to those who, without the clinical intervention of the MHP, are likely to have inadequate prenatal care until the time of delivery.

These results indicate that mobile prenatal programs provided at no cost to patients can be valuable in addressing inequitable obstetric outcomes for uninsured patients and others who face barriers to accessing prenatal services. Longitudinal studies aggregating delivery outcomes, as well as other obstetrical care metrics, amongst a network of mobile prenatal clinics would combat small sample sizes and further elucidate the impact of mobile healthcare on maternal–neonatal health.

## Figures and Tables

**Table 1:** Demographic information for the 331 patients provided care at University of Arizona Mobile Health Program (MHP) between 2019 and 2024.

Characteristic	N = 331
Race, n (%)	
White	42 (12.7%)
Black or African American	13 (3.9%)
Other	3 (0.9%)
Missing	273 (82.5%)
Ethnicity, n (%)	
Hispanic or Latino	203 (61.3%)
Not Hispanic or Latino	29 (8.8%)
Missing	99 (29.9%)
Age (years), mean (SD)	30.7 (5.8)
Pre-existing medical condition, n (%)	155 (46.8%)
Pre-existing mental health condition, n (%)	8 (2.4%)
No. of Visits, mean (SD)	8.6 (4.9)

## Data Availability

The data used for analysis for this article contain protected health information and are not readily available due to privacy and confidentiality regulations. De-identified data may be made available from the corresponding author upon reasonable request with appropriate institutional approvals.

## Abbreviations

The following abbreviations are used in this manuscript:

CS	Cesarean Section
MHP	Mobile Health Program
EMR	Electronic Medical Record
